# Contraceptive Use in Women Enrolled into Preventive HIV Vaccine Trials: Experience from a Phase I/II Trial in East Africa

**DOI:** 10.1371/journal.pone.0005164

**Published:** 2009-04-10

**Authors:** Hannah Kibuuka, David Guwatudde, Robert Kimutai, Lucas Maganga, Leonard Maboko, Cecilia Watyema, Fredrick Sawe, Douglas Shaffer, Dickson Matsiko, Monica Millard, Nelson Michael, Fred Wabwire-Mangen, Merlin Robb

**Affiliations:** 1 Makerere University-Walter Reed Project, Kampala, Uganda; 2 Makerere University School of Public Health , Kampala, Uganda; 3 Walter Reed Project, Kericho, Kenya; 4 Mbeya Medical Research Programme, Mbeya, Tanzania; 5 US Military HIV Research Program, Rockville, Maryland, United States of America; Yale University, United States of America

## Abstract

**Background:**

HIV vaccine trials generally require that pregnant women are excluded from participation, and contraceptive methods must be used to prevent pregnancy during the trial. However, access to quality services and misconceptions associated with contraceptive methods may impact on their effective use in developing countries. We describe the pattern of contraceptive use in a multi-site phase I/IIa HIV Vaccine trial in East Africa (Uganda, Kenya and Tanzania) and factors that may have influenced their use during the trial.

**Methods:**

Pregnancy prevention counseling was provided to female participants during informed consent process and at each study visit. Participants' methods of contraception used were documented. Methods of contraceptives were provided on site. Pregnancy testing was done at designated visits during the trial. Obstacles to contraceptive use were identified and addressed at each visit.

**Results:**

Overall, 103 (31.8%) of a total of 324 enrolled volunteers were females. Female participants were generally young with a mean age of 29(±7.2), married (49.5%) and had less than high school education (62.1%). Hormonal contraceptives were the most common method of contraception (58.3%) followed by condom use (22.3%). The distribution of methods of contraception among the three sites was similar except for more condom use and less abstinence in Uganda. The majority of women (85.4%) reported to contraceptive use prior to screening. The reasons for not using contraception included access to quality services, insufficient knowledge of certain methods, and misconceptions.

**Conclusion:**

Although hormonal contraceptives were frequently used by females participating in the vaccine trial, misconceptions and their incorrect use might have led to inconsistent use resulting in undesired pregnancies. The study underscores the need for an integrated approach to pregnancy prevention counseling during HIV vaccine trials.

**Trial Registration:**

ClinicalTrials.gov NCT00123968

## Introduction

Women in Sub-Saharan Africa carry a disproportionately large burden of the HIV infection. Young women (15–24 yrs) are four times more likely to be HIV infected than are young men [1]. Despite the risk for HIV infection, women are less likely to benefit from existing behavioral and biomedical HIV prevention interventions due to social, cultural and economic barriers [2]. Developing an effective woman-controlled intervention like a vaccine is therefore a high priority to reduce their vulnerability to HIV. It is thus of utmost importance that women in Sub Saharan Africa participate in HIV vaccine trials since they stand to benefit most from an effective HIV vaccine. In addition, their participation will not only accelerate HIV vaccine development but also allow for valid gender-specific assessment of efficacy and safety [3], [4].

However, there are several challenges to female participation in these vaccine trials [5]. One of the challenges has arisen from the requirement that women of childbearing potential use effective contraception during their participation in HIV prevention trials [6], [7].

There have been efforts to advance HIV vaccine research globally and particularly in Sub Saharan Africa [8]. Since the first HIV vaccine trial in Africa was conducted in Uganda in 1999, several HIV vaccine trials have been conducted in the region [7], [9]. Female participation in most of these trials, particularly in East Africa, has been low [10]. In the first US Military HIV Research Program (USMHRP) phase I HIV vaccine trial to be conducted in East Africa, where both men and women presented for enrollment screening in equal numbers, there was only 13% female enrollment despite equivalent rates of eligibility.

HIV vaccine trials require that women of reproductive age use contraception during the trial since little or no human data exist regarding vaccine safety in pregnancy. Although there has been an increase in contraceptive use in Sub-Saharan Africa, their use remains relatively low compared to other parts of the world[11], [12], [13]. Past studies have shown that 2–20% of women in Sub–Saharan Africa use contraception covertly [14], [15]. However recent literature on this subject is scanty. There are various difficulties women face in accessing contraception. These include lack of social support (including spousal support), low male involvement, existing myths and fear of method side effects, low socio-economic status, high levels of illiteracy and scarcity of family planning services[7], [16], [17]. These same difficulties may impact contraceptive use in vaccine trials further limiting the number of female participants in these trials.

Assessing the level and factors influencing use of contraceptives during HIV vaccine trials provides vital information for contraceptive and pregnancy prevention counseling for purposes of recruitment and retention of females in these trials. We describe the pattern of contraceptive use at enrollment and factors that might have influenced their use, among 103 female participants in a phase I/IIa HIV Vaccine trial (RV 172) conducted at USMHRP sites in East Africa, May 2006 to August 2007.

## Methods

### Study overview

RV 172 was a phase I/IIa HIV clinical trial to evaluate the safety and immnunogenicity of a multiclade HIV DNA plasmid vaccine VRC-HIVDNA016-00-VP, boosted by a Multiclade HIV-1 Recombinant Adenovirus-5 vector vaccine, VRC-HIVADV014-00-VP, in uninfected adult volunteers in East Africa. Participants were enrolled at the three USMHRP supported East Africa HIV vaccine research sites. The Makerere University-Walter Reed Project (MUWRP), in Kampala, Uganda, recruited participants from the urban and semi-urban populations within a radius of 20 kilometers of Kampala city. The Walter Reed Project-Kericho (WRP-Kericho), located on the grounds of Kericho District Hospital in Kericho Kenya, recruited from rural and urban populations within a radius of 20 kilometers from Kericho town. The Mbeya Medical Research Council (MMRP) located on the Mbeya Hospital campus recruited participants from semi-urban populations within a radius of 10km from Mbeya town in Tanzania.

At all the three study sites, the informed consent process was conducted over 2–7 days and consisted of a general information session where participants were provided with general information on HIV vaccine research, a briefing session to provide study details and a one-on-one informed consent session to obtain written approval to participate in study.

### Pregnancy prevention counseling and contraceptive access and use

At all the three study sites, detailed information about study related pregnancy risks was given during briefing and the informed consent sessions. Pregnant women, breast feeding women and those desiring to become pregnant during the study were excluded from participation. During the informed consent session and at each clinic visit, study staff provided information on methods of contraception, discussed individual contraceptive choices and provided pregnancy prevention counseling.

Each participant confirmed either verbally or by documentation to have used contraception for at least 21 days prior to enrollment. An agreed on method of contraception for each participant, was documented as part of eligibility. For the majority of participants (73.8%), the information on contraceptive use was based on self report although when possible efforts were made to verify the information using family planning cards or medical records.

In an attempt to enhance access to various effective contraceptive methods, arrangements were made with family planning providers to supply contraceptives that were not being provided at the study sites. MUWRP, Uganda offered male condoms onsite and referral to an offsite family planning service located 1 km from the clinic, which provided a range of contraceptives. MMRP, Tanzania and WRP-Kericho-Kenya are located on hospital premises where family planning services are provided. MMRP clinic offered male condoms only onsite while WRP-Kericho, Kenya offered male condoms, oral contraceptives and injectable contraceptives on site.

Participants had options to access contraceptives from the study sites, or from their usual service providers and/or from the offsite family planning providers. Contraceptive methods reported in this study were those used by participants at enrollment. For the majority of participants, there was no difference between contraceptive method used at enrollment and during the study. Only seven (6.8%) participants changed the methods of contraception used during the study and these changes were mostly late in the study and had no impact on the analysis for this article.

Blood pregnancy tests were done during screening, before each vaccination, and 2–6 weeks following the last vaccination. Pregnancy during the study vaccination period resulted into withdrawal from the vaccination schedule.

Obstacles to contraceptive use identified by voluntary disclosure were addressed during individual pregnancy prevention counseling sessions' at each study site. To assist participants further understand the different contraceptive methods used and to explore barriers to contraceptive use and how best to address the obstacles, a focus group discussion was held with participants at the Uganda site. A pictorial family planning chart was used to help participants better understand contraceptive use and enhance individual and group pregnancy prevention counseling. The focus group discussion was conducted later during the follow-up phase of the study.

The study received scientific and ethical approvals from the Walter Reed Army Institute of Research Human Use Research Committee (WRAIR HURC) and from the National AIDS Research Committee (Uganda), Kenya Medical Research Institute Ethical Review Committee (Kenya) and the Mbeya Ethical Review Committee and the National Ethical Committee/Medical Research Coordinating Committee (Tanzania).

### Data analysis

Frequency distributions and proportions were used to describe participants' prior contraceptive use at the study sites. The proportions of participants using the different types of contraceptives and the pattern of use at the three study sites are described by their social and demographic characteristics.

Binary logistic regression analysis was used to identify factors including social and demographic characteristics, associated with hormonal contraceptive use. For this analysis, adjusted Odds Ratios (ORs) with the corresponding 95% confidence intervals (95% CI) are reported. The data was analyzed using SAS software.

## Results

Out of the 324 participants enrolled in the RV172 clinical trial, 103 (31.8%) were females. A higher proportion of females, 35 out of 60(58 %) were enrolled at MMRP, Tanzania, compared to 38 out of 144(26%) at MUWRP, Uganda and 30 out of 120(25%) at WRP-Kericho, Kenya. The female participants were generally young with a median age of 29 years (18–47). About half (49.5%) of all females were married. The majority of female participants were self employed or had paid employment (56.3%), and had no education or less than high school education (62.1%). Female participants at the Tanzanian site were less educated with 94.3% not achieving a high school level of education compared to 42.1% and 50% for Uganda and Kenya respectively(see Table 1).

**Table 1:** Demographic Characteristics of Female Study Participants by Site

Characteristics	Category	All sites	MUWRP Uganda	WRP-Kericho Kenya	MMRP Tanzania
		n (%)	n (%)	n (%)	n (%)
All participants:	-	103	38	30	35
Education:	None/Unable to read	4 (3.9)	2 (5.3)	1 (3.3)	1 (2.9)
	Less than high school /Primary	60 (58.3)	14 (36.8)	14 (46.7)	32 (91.4)
	High school /Secondary	27 (26.2)	18 (47.4)	7 (23.3)	2 (5.7)
	Tertially education/Degree	12 (11.7)	4 (10.5)	8 (26.7)	0 (0.0)
Marital Status:	Married	51 (49.5)	18 (47.4)	14 (46.7)	19 (54.3)
	Single	38 (36.9)	16 (42.1)	12 (40.0)	10 (28.6)
	Separated/Widowed	14 (13.6)	4 (10.5)	4 (13.3)	6 (17.1)
Employment Status:	Employed/Self employed	58 (56.3)	19 (50.0)	22 (73.3)	17 (48.6)
	Unemployed	38 (36.9)	16 (42.1)	7 (23.3)	15 (42.9)
	Student	7 (6.8)	3 (7.9)	1 (3.3)	3 (8.6)
Median Age (range):	-	29 (18–47)	29 (19–47)	29 (20–43)	30 (18–47)

Contraceptive methods used at the three sites included hormonal contraceptives, male condoms, Bilateral Tubal Ligation (BTL) and abstinence. Hormonal contraceptives were the most commonly used accounting for 55.3% at MUWRP, Uganda, 53.3% at WRP-Kericho, Kenya and 65.7% at MMRP, Tanzania (Table 2). Depo-Provera was more commonly used (34%) compared to other hormonal contraceptives (24.3%). For the other forms of contraception, there was higher condom use at MUWRP, Uganda (34.2%) compared to WRP-Kericho, Kenya (10.0%) and MMRP, Tanzania (20.0%) whereas abstinence and BTL were higher at WRP-Kericho, Kenya (36.7%) compared to MUWRP, Uganda (10.5%), and MMRP, Tanzania (14.3%).

**Table 2:** Duration of Contraceptive use prior to Screening, Contraceptive Methods Used at enrollment and Pregnancies Reported

Characteristic	Category	All sites	MUWRP Uganda	WRP-Kericho Kenya	MMRP Tanzania
		n(%)	n (%)	n (%)	n (%)
All participants:		103	38	30	35
Duration of contraceptive use prior to screening:	≤1 year	20 (19.4)	4 (10.5)	11 (36.7)	5 (14.3)
	>1 year	33 (32.0)	11 (28.9)	7 (23.3)	15 (42.9)
	Unknown	35 (34.0)	17 (44.7)	9 (30.0)	9 (25.7)
	None used	15 (14.6)	6 (15.8)	3 (10.0)	6 (17.1)
Contraceptive at enrollment:	Hormonal	60 (58.3)	21 (55.3)	16 (53.3)	23 (65.7)
	Condoms	23 (22.3)	13 (34.2)	3 (10.0)	7 (20.0)
	Other (Abstinence & BTL)	20 (19.4)	4 (10.5)	11 (36.7)	5 (14.3)
Pregnancies (rates):	-	9 (8.7)	5 (13.2)	1 (3.3)	3 (8.6)

The majority of the female participants reported to have been using contraception prior to being screened for the study; 19.4% reported to have been using contraception for one year or less, 32.0% for more than one year, whereas 34.0% reported to have used contraception for undetermined period of time. Only 14.6% of the female participants initiated contraception for purposes of participating in the trial.

The pattern of use was different among married participants compared to single/ separated/widowed participants (Table 3). Married women were more likely to use hormonal contraception, OR 3.3 (1.34–7.93) and less likely to use male condoms, OR 0.3 (012–0.97). When adjusted for marital status, age, employment status, education level attained and country were not associated with male condom or hormonal contraceptive use (Table 4 and 5) except for significantly less male condom use by Kenyan participants, OR 0.2 (0.005–0.82).

**Table 3:** Pattern of Contraceptive Use at Enrolment by Selected Demographic Factors (All Sites)

Demographic factor	Category	Total # on Contraception	# Using Hormonal Contraceptives (%)	# Using Condom (%)	# Using Other Contraceptives (%)
Occupation:	Employed	58	30 (51.7)	13 (22.4)	15 (25.9)
	Unemployed/student	45	30 (66.7)	10 (22.2)	5 (11.1)
Marital status:	Married	51	37 (72.5)	8 (15.7)	6 (11.8)
	Single/Separated/widowed	52	23 (44.2)	15 (28.8)	14 (26.9)
Age:	<24	33	18 (54.5)	9 (27.3)	6 (18.2)
	25–34	44	28 (63.6)	11 (25.0)	5 (11.4)
	>35	26	14 (53.8)	3 (11.5)	9 (34.6)
Education:	Less than High School	64	40 (62.5)	14 (21.9)	10 (15.6)
	High School and above	39	20 (51.3)	9 (23.1)	10 (25.6)

**Table 4:** Use of Hormonal Contraceptives by Participant Characteristics*

Characteristics	Category	-n-	#Using Hormonal Contraceptives (%)	Adjusted OR [95%CI]
All participants:	-	103	60 (58.3)	-
Marital Status:	Single	38	17 (44.8)	1.0
	Married	51	37(72.5)	3.3 [1.34–7.93]**
	Separated/Divorced	14	6 (42.9)	0.9 [0.27–3.19]
Age (yrs):	18–30	64	38 (59.4)	1.0
	31–49	39	22 (56.4)	0.7 [0.30–1.71]
	All ages	103	60 (58.3)	0.97 [0.91–1.03]
Education:	Primary or none	64	40 (62.5)	1.0
	Secondary or higher	39	20 (51.3)	0.8 [0.35–1.97]
Occupation:	Employed	58	30 (51.7)	1.0
	Un-employed	38	26 (68.4)	1.4 [0.54–3.57]
	Student	7	4 (57.1)	1.7 [0.31–8.91]
Country:	Uganda	38	21 (55.3)	1.0
	Kenya	30	16 (53.3)	0.9 [0.34–2.54]
	Tanzania	35	23 (65.7)	1.5 [0.56–4.05]

* Response variable coded as: 0 = Did not use hormonal contraceptives, 1 = Used hormonal contraceptives.

** Significant observation.

**Table 5:** Previous Condom Use as Contraception by Participant Characteristics*

Characteristics	Category	-n-	#Using Condom (%)	Adjusted OR [95%CI]
All participants:	-	103	23 (22.3)	-
Marital Status:	Single	38	13 (34.2)	1.0
	Married	51	8 (15.7)	0.3 [0.12–0.97]**
	Separated/Divorced	14	2 (14.3)	0.3 [0.06–1.74]
Age (yrs):	18–30	64	16 (25.0)	1.0
	31–49	39	7 (17.9)	0.8 [0.27–2.28]
	All ages	103	23 (22.3)	0.99 [0.92–1.07]
Education:	Primary or none	64	14 (21.9)	1.0
	Secondary or higher	39	9 (23.1)	0.7 [0.20–2.20]
Occupation:	Employed	58	13 (22.4)	1.0
	Un-employed	38	7 (18.4)	0.7 [0.23–2.35]
	Student	7	3 (42.9)	1.4 [0.23–8.14]
Country:	Uganda	38	13 (34.2)	1.0
	Kenya	30	3 (10.0)	0.2 [0.05–0.82]**
	Tanzania	35	7 (20.0)	0.5 [0.18–1.63]

* Response variable coded as: 0 = Did not use condom for contraception, 1 = Used condoms as contraception.

** Significant observation.

Nine pregnancies were diagnosed at the three sites (Table 2). Uganda reported more pregnancies (13.2%) compared to Kenya (3.3%) and Tanzania (8.6%).

Seven out of the nine participants (77.8%) who became pregnant reported using hormonal contraceptives. Five participants on hormonal contraception reported using Depo-Provera. Of these participants, one reported to having agreed to use Depo-Provera because the study required contraceptive use although she had no intention of using it. Two participants had documentation of duly completed family planning cards and maintained that they had received all injections as required. One participant was requested to use dual methods during the first two weeks of Depo-Provera injection but disregarded the advice while the last participant delayed re-administration of the Depo-Provera injection. The other participants on hormonal contraceptives were using oral contraceptive pills.

Two participants relying on male condoms became pregnant. One pregnancy resulted from inconsistent condom use while the second pregnancy was due to a participant's change of mind about contraception in order to maintain a new relationship.

Barriers to contraceptive use identified during individual counseling and the focus group discussions included, insufficient knowledge on use of chosen contraceptive methods, lack of partner support, and myths and misconceptions surrounding contraceptive use. One participant on Depo-Provera reported intermittent use due to a belief that injections are not required when experiencing Depo-Provera induced amenorrhea. Another participant on oral contraceptives was using them as morning after pill.

Other common misconceptions included contraceptive use being a cause of congenital abnormalities, infertility, and loss of libido, decreasing in vaginal fluids and delaying conception regardless of type of contraception. Some nulliparous participants chose to use condoms over hormonal contraception because of fear of loss of fertility.

The majority of participants opted to continue with their family planning service provider rather than be referred to a new service provider. At MUWRP, Uganda and MMRP, Tanzania, the two sites that did not offer hormonal contraceptives on site, 43 out of 44 participants (97.7%) on hormonal contraceptives continued accessing contraceptives from their usual family planning service providers. Only one participant switched to accessing contraceptives from a new service provider. The main reasons why participants preferred to continue with their usual family planning service providers were that some participants had been getting Depo-Provera injections for a number of years and did not find it necessary to change given that the study was temporary and other participants resided near the service providers and therefore found it more convenient to continue with these providers. However, this created another barrier since availability and accessibility to quality contraceptives at outside service providers could not be ensured.

## Discussion

In this study, contraceptive use and effectiveness were assessed in the context of participation in an experimental vaccine trial which required use of effective birth control methods. The observations may not therefore generalize to the population of women as a whole but are relevant to conduct of experimental studies of vaccines and potentially other products. The majority of participants used hormonal contraceptives at all the three study sites. The choice to use hormonal contraceptives particularly Depo-Provera is perhaps a reflection of the pattern of contraceptive use in sub-Saharan Africa as described by Seiber et al (2007) [11]. Marital status significantly influenced the pattern of contraceptive use. Married participants preferred to use hormonal contraceptives, which differs from what is previously reported [18]. The use of Depo-Provera would be ideal for clinical trials and offers several benefits including convenience, covert use and therefore a female controlled intervention with wide accessibility [19]. However, the majority of participants who became pregnant reported using hormonal contraception, including five of who reported using Depo-Provera. This demonstrates the existing challenge to effective contraception or self report of contraceptive use in clinical trials. Since hormonal contraceptives were not routinely offered on site and most participants desired to continue with their family planning service provider where their availability, accessibility or quality could not be assured, it is possible that this limited their optimal utilization. It also makes it difficult for investigators to accurately verify information given by participants about contraceptive use. This could suggest better utilization if contraceptives are provided on site or establishing a clear referral mechanism to a reliable family planning provider to ensure access to quality methods and their consistent use. It could also be important to use objective measures such as a requirement to present a family planning card, to assess consistent contraceptive use.

A pregnancy rate of 8.9% in the study is within range of 3–15% risk of pregnancy among typical hormonal and condom users in the first year [20]. A further reduction in number of pregnancies in phase I/II vaccine studies would be desirable and calls for an integrated approach to pregnancy prevention including strategies to identify and address factors that inhibit ideal use.

It is possible to conclude that the higher pregnancy rates at MUWRP, Uganda is a reflection of Uganda's high fertility rate of 6.6 compared to 5.0 for Kenya and 5.3 for Tanzania[21] however, this is less likely since those that desired pregnancy were excluded and ongoing pregnancy prevention counseling was given during the trial. Moreover, many women were already on contraception prior to study screening. Changes to women's desire to have children during the study could also be a possible explanation.

Although practiced in a few participants, abstinence alone was a reliable method when chosen as an option, but investigators need to regularly review participant's adherence to this method and intervene with an alternative promptly when sexual activity is initiated or resumed. Bilateral tubal ligation (BTL), another effective method was rarely used given the average age of participants. The small numbers involved in the sub-analysis however limit the generalizability of these results.

Myths and misconceptions to contraceptive use continue to be barriers to their effective use. Although their influence was not measured in quantitative terms, it is possible that they impacted contraceptive use. There is a need to identify and address them on an ongoing basis during pregnancy prevention counseling. We found group discussions to be a better forum for disclosing misconceptions regarding contraceptive methods than individual counseling, which could probably be due to social desirability that can occur during individual counseling, since participants may not want to be viewed as non study compliant. In addition, group discussion allows debate and expression on issues outside the context of a study.

### Conclusion

The study reflects the contraceptive experience and effectiveness among women who participated in an HIV vaccine trial which required contraception during the study. Only a minority of women entering the vaccine study initiated contraception at the time of study entry. Obstacles to successful contraception may include misconceptions about contraceptive methods among volunteers and ineffective administration of methods by participants or family planning providers. Mitigation of these obstacles may include more intensive and recurring discussion of contraceptive technologies to improve participant knowledge and acceptance. In addition, integrating provision of family planning services with research activities may ensure appropriate use of quality contraceptive products.
